# Thymidine phosphorylase/platelet-derived endothelial cell growth factor is upregulated in advanced solid types of gastric cancer

**DOI:** 10.1038/sj.bjc.6690182

**Published:** 1999-03

**Authors:** T Yoshikawa, K Suzuki, O Kobayashi, M Sairenji, H Motohashi, A Tsuburaya, Y Nakamura, A Shimizu, S Yanoma, Y Noguchi

**Affiliations:** The Third Department of Surgery, Kanagawa Cancer Center, Yokohama, 241-0815, Japan; The First Department of Pathology, Kanagawa Cancer Center, Yokohama, 241-0815, Japan; The Second Department of Biochemistry, Kanagawa Cancer Center, Yokohama, 241-0815, Japan; The First Department of Surgery, Yokohama City University, School of Medicine, Asahi-Ku, Yokohama, 241-0815, Japan

**Keywords:** thymidine phosphorylase, platelet-derived endothelial cell growth factor, gastric carcinoma

## Abstract

Previous studies demonstrated that the immunohistochemical expression of thymidine phosphorylase (dThdPase) was related with distant metastasis and disease progression. In this study we investigated the production of dThdPase/platelet-derived endothelial cell growth factor in gastric cancer quantitatively. In a total of 75 tumour tissues and 60 normal gastric mucosa specimens, dThdPase protein concentrations were determined by ELISA. The amount of dThdPase was significantly higher in the tumour tissue than in the normal tissue. Intratumoural dThdPase concentrations were significantly higher in Borrmann types I and II macroscopically, in poorly differentiated and solid type histologically, in the medullary type of the tumour stroma, and in the tumour-invading serosa. In the medullary type of the amount of tumour stroma, protein levels of dThdPase were positively correlated with the vertical diameter of the tumour (*r* = 0.580, *P* = 0.019). By immunohistochemical study, dThdPase expression on tumour cells was observed in all seven specimens with high dThdPase protein levels, but not in all 14 cases with low dThdPase protein levels (*P* < 0.05). In summary, these data indicated that dThdPase is up-regulated in advanced solid types of gastric cancer, suggesting that dThdPase production in carcinoma cells might be induced by the microenvironment. © 1999 Cancer Research Campaign


*British Journal of Cancer* (1999) **79**(7/8), 1145–1150
© 1999 Cancer Research Campaign
Article no. bjoc.1998.0182

### Thymidine phosphorylase/platelet-derived endothelial cell growth factor is upregulated in advanced solid types of gastric cancer

**T Yoshikawa[1], K Suzuki[1], O Kobayashi[1], M Sairenji[1], H Motohashi[1], A Tsuburaya[1], Y Nakamura[2], A Shimizu[2], S Yanoma[3] and Y Noguchi[4]**

[1]The Third Department of Surgery, [2]The First Department of Pathology and [3]The Second Department of Biochemistry, Kanagawa Cancer Center, and [4]The First Department of Surgery, Yokohama City University, School of Medicine, 1-1-2 Nakao, Asahi-Ku, Yokohama 241-0815, Japan

**Summary** Previous studies demonstrated that the immunohistochemical expression of thymidine phosphorylase (dThdPase) was related with distant metastasis and disease progression. In this study we investigated the production of dThdPase/platelet-derived endothelial cell growth factor in gastric cancer quantitatively. In a total of 75 tumour tissues and 60 normal gastric mucosa specimens, dThdPase protein concentrations were determined by ELISA. The amount of dThdPase was significantly higher in the tumour tissue than in the normal tissue. Intratumoural dThdPase concentrations were significantly higher in Borrmann types I and II macroscopically, in poorly differentiated and solid type histologically, in the medullary type of the tumour stroma, and in the tumour-invading serosa. In the medullary type of the amount of tumour stroma, protein levels of dThdPase were positively correlated with the vertical diameter of the tumour ($r = 0.580$, $P = 0.019$). By immunohistochemical study, dThdPase expression on tumour cells was observed in all seven specimens with high dThdPase protein levels, but not in all 14 cases with low dThdPase protein levels ($P < 0.05$). In summary, these data indicated that dThdPase is up-regulated in advanced solid types of gastric cancer, suggesting that dThdPase production in carcinoma cells might be induced by the microenvironment.

**Keywords:** thymidine phosphorylase; platelet-derived endothelial cell growth factor; gastric carcinoma

Thymidine phosphorylase (dThdPase) is an enzyme that is involved in pyrimidine nucleoside metabolism. It catalyses the reversible phosphorolysis of thymidine, deoxyuridine and their analogues to their respective bases and 2-deoxyribose-1-phosphate (Cook et al, 1979; Eda et al, 1993). Using dThdPase, 5′-deoxy-5-fluorouridine (5′-DFUR), a prodrug of 5-fluorouracil (5-FU), is converted to 5-FU which is an anti-tumour drug used to treat a variety of neoplastic diseases such as gastric carcinoma (Ishitsuka et al, 1980; Miwa et al, 1987). As the expression of dThdPase is higher in tumours than in normal tissue (Kono et al, 1983; Nio et al, 1992), 5′-DFUR is used for several kinds of human tumours. However, to ensure suitable administration of 5′-DFUR, it is important to select the patients whose tumour tissue shows a much higher content of dThdPase.

Recently, it was reported that dThdPase is identical to platelet-derived endothelial cell growth factor (PD-ECGF) (Ishikawa et al, 1989; Furukawa et al, 1992). PD-ECGF stimulates chemotaxis of endothelial cells in vitro and has an angiogenic activity in vivo (Ishikawa et al, 1989; Miyazono et al, 1987). Experimental evidence has shown that angiogenesis plays a significant role in tumour growth and metastasis (Folkman, 1990; Folkman and Klagsbrun, 1987).

Thus far, the expression of dThdPase has been examined in a variety of types of neoplasm by immunohistochemistry, and its relations with prognosis, distant metastasis and disease progression have been discussed (Maeda et al, 1996; Takebayashi et al., 1996a). However, neither the characteristics of tumours with much higher protein levels of dThdPase nor the regulatory mechanisms of dThdPase production in gastric cancer has been clarified.

In this study, in order to quantitatively assess the clinicopathological factors related to dThdPase and to elucidate the mechanisms of dThdPase induction, the intratumoural protein levels of dThdPase were determined by enzyme-linked immunosorbent assay (ELISA) in 75 gastric carcinoma tissues. For localization of dThdPase in cancer tissue, immunohistochemical studies were also performed.

*Received 21 April 1998*
*Revised 15 June 1998*
*Accepted 13 July 1998*

*Correspondence to:* T Yoshikawa

### MATERIALS AND METHODS

### Patients

Tissue samples were obtained from 75 gastric cancer patients who underwent surgical resection at Kanagawa Cancer Center between August 1996 and February 1998. All patients were primary, except one patient with recurrent nodules of peritoneal dissemination. Classification of the resected gastric cancer tissues and pathological diagnosis were carried out according to the General Rules for the Gastric Cancer Study in Surgery and Pathology in Japan (Japanese Research Society for Gastric Cancer, 1995). Macroscopic types, based on Borrmann's classification, were as follows:

- type 0 – early gastric cancer
- types 1, 2, 3 and 4 – Borrmann types I, II, III and IV advanced gastric cancer
- type 5 – unclassified advanced gastric cancer (Japanese Research Society for Gastric Cancer, 1995).

### Preparations of tissue samples

Primary tumour tissues in 74 primary gastric cancer patients and a disseminated nodule in one recurrent patient were obtained during surgery. Normal gastric mucosa specimens were also resected in 60 primary tumour patients. Tissue samples were immediately frozen after the removal and stored at –80°C until processing. The frozen tissue samples were homogenized with phosphate-buffered saline (PBS) and centrifuged at 8000 ***g*** for 20 min. The supernatants were then used for the detection of the amount of dThdPase. The protein content was determined by DC protein assay (Bio-Rad, Hercules, CA, USA).

### dThdPase assay

The amount of dThdPase protein was quantitated with ELISA as described (Nishida et al, 1996). Ninety-six-well microtiter plates were coated with 10 μg/ml of anti-human dThdPase monoclonal antibody 104B (Nippon Roche Research Center, Kanagawa, Japan) in PBS solution and then incubated with blocking solution containing skim milk. This antibody was prepared by using as an antigen human dThdPase purified from human colon cancer HCT116. The characterization of the antibody has been reported previously (Nishida et al, 1996). For the assay, 50 μl of properly diluted samples and serially diluted dThdPase (standards) were added to the wells and incubated for 2 h at 37°C, then washed with 0.05% Tween 20 in PBS and incubated with dThdPase monoclonal antibody 232-1. After washing the wells four times, 50 μl of anti-mouse IgG antiserum conjugated with peroxidase was added to the wells and incubated for 1 h at room temperature. Wells were washed four times, and then the enzyme reaction was carried out at 37°C for 1 h with a substrate solution containing 3,3′,5,5′-tetramethylbenzidine (TMB) and hydrogen peroxide (TMB microwell peroxidase substrate system, KPL). The reaction was stopped by adding phosphoric acid and the absorbance at 450 nm was measured with a microplate reader. dThdPase contents were adjusted by the whole protein concentrations and expressed as unit/mg protein (U/mg protein).

### Immunohistochemistry

For localization of dThdPase in gastric cancer tissue, immunohistochemical studies were performed on deparaffinized sections by the peroxidase-labelled streptavidin–biotin method using anti-dThdPase monoclonal antibody 654-1 (Nishida et al, 1996), provided by Nippon Roche Research Center, in seven tumour specimens with extremely high protein levels of dThdPase (≤ 200 U/mg protein) and 14 tumour tissues with low protein levels of dThdPase (< 70 U/mg protein). The clinicopathological characteristics of these cases are shown in Table 1.

Briefly, the sections were treated with skim milk in PBS at room temperature and incubated with the anti-dThdPase mouse monoclonal antibody (1 μg/ml) overnight at 4°C. After washing with PBS, the sections were further incubated with biotinylated anti-mouse IgG for 30 min at room temperature. Thereafter, the sections were treated with 1 mg/ml periodic acid in PBS for 10 min to block endogenous peroxidase activity. After being washed again with PBS, the sections were incubated with

**Table 1** Clinicopathological characteristics in 21 cases selected for immunohistochemistry

| Case | Macroscopic type | Vertical diameter (cm) | Histological type | Amount of tumour stroma | Depth of invasion | Venous invasion | dThdPase contents (U/mg protein) |
|---|---|---|---|---|---|---|---|
| *High levels of dThdPase protein (≥ 200 U/mg protein)* | | | | | | | |
| 1 | 1 | 2.2 | Poorly differentiated and solid | Medullary | SE | Positive | 210.0 |
| 2 | 3 | 2.5 | Moderately differentiated tubular | Medullary | SE | Negative | 217.4 |
| 3 | 3 | 1.4 | Mucinous | Intermediate | SE | Negative | 262.5 |
| 4 | 1 | 1.0 | Moderately differentiated tubular | Intermediate | SE | Positive | 411.9 |
| 5 | 2 | 1.4 | Moderately differentiated tubular | Medullary | SI | Negative | 210.3 |
| 6 | 2 | 1.0 | Poorly differentiated and solid | Medullary | SE | Negative | 310.8 |
| 7[a] | 2 | 2.0 | Poorly differentiated and solid | Medullary | SE | Positive | 459.2 |
| *Low levels of dThdPase protein (< 70 U/mg protein)* | | | | | | | |
| 8 | 4 | 2.3 | Poorly differentiated and non-solid | Scirrhous | SE | Negative | 37.2 |
| 9 | 4 | 1.2 | Poorly differentiated and non-solid | Scirrhous | SE | Positive | 65.9 |
| 10[b] | 4 | 2.0 | Poorly differentiated and non-solid | Scirrhous | SE | Negative | 42.4 |
| 11 | 4 | 1.6 | Poorly differentiated and non-solid | Scirrhous | SE | Negative | 69.9 |
| 12 | 4 | 2.3 | Mucinous | Scirrhous | SE | Positive | 58.3 |
| 13 | 4 | 1.0 | Poorly differentiated and non-solid | Scirrhous | SE | Negative | 33.1 |
| 14 | 4 | 2.1 | Poorly differentiated and non-solid | Scirrhous | SE | Positive | 56.9 |
| 15 | 3 | 1.8 | Poorly differentiated and solid | Intermediate | SE | Negative | 53.0 |
| 16 | 2 | 0.8 | Poorly differentiated and solid | Intermediate | SE | Positive | 38.6 |
| 17 | 3 | 1.4 | Mucinous | Intermediate | SE | Positive | 67.5 |
| 18 | 3 | 1.2 | Moderately differentiated tubular | Intermediate | SE | Positive | 57.4 |
| 19 | 0 | ≤ 0.5 | Moderately differentiated tubular | Medullary | M | Negative | 48.7 |
| 20 | 0 | ≤ 0.5 | Well differentiated tubular | Medullary | M | Negative | 68.6 |
| 21 | 0 | ≤ 0.5 | Moderately differentiated | Medullary | SM | Negative | 62.3 |

[a]Immunohistochemical findings in case 7, shown in Figure 4. [b]Immunohistochemical findings in case 10, shown in Figure 5. M, mucosal neoplastic involvement; SM, submucosal neoplastic involvement; SE, serosal involvement; SI, serosal involvement with directly infiltrating other organs beyond serosa. Macroscopic types are based on Borrmann's classification as follows: type O, early gastric cancer; types 1, 2, 3 and 4, Borrmann's types I, II, III and IV advanced gastric cancer; type 5, unclassified advanced gastric cancer.

 © Cancer Research Campaign 1999

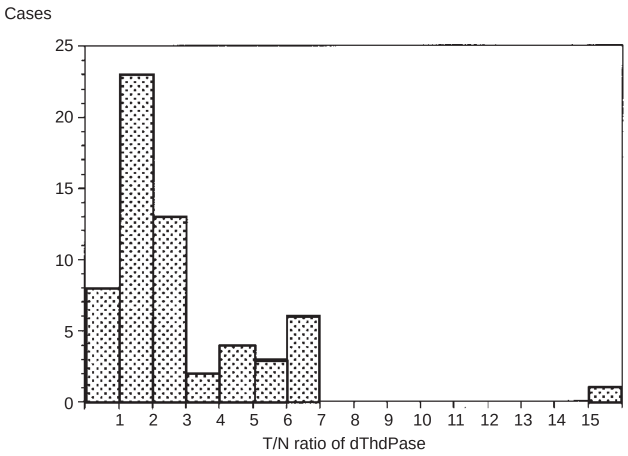

**Figure 1** Distribution of tumour–normal (T/N) ratio of thymidine phosphorylase protein concentrations determined by ELISA

avidin–biotin complex (Vectastain ABC kit: Vector, Burlingame, CA, USA) for 30 min at room temperature and developed with 1 mg/ml diaminobenzidine tetrahydrochloride in PBS containing 0.03% hydrogen peroxide. The sections were also counterstained with methyl green and mounted.

We decided that the tumour cells should be regarded as dThdPase-positive when more than 50% of the tumour cells were stained. Infiltrating cells invading tumour tissue were also regarded as dThdPase-positive when more than 50% of the infiltrating cells were stained.

### Statistical analysis

Comparisons between the two groups were performed by paired and unpaired Student's *t*-test and $\chi^2$ method. For more than three groups, one-way analysis of variance (ANOVA) and Duncan's multiple range test were used. Correlations were evaluated by Spearman's test. $P < 0.05$ was considered to indicate statistical significance. Data were expressed as mean ± SD.

### RESULTS

The amount of dThdPase was significantly higher in the tumour tissue than in the normal tissue by paired Student's *t*-test (89.3 ± 86.6 U/mg protein, 36.8 ± 24.3 U/mg protein, respectively). The tumour–normal (T/N) ratios of dThdPase contents were more than 1.0 in 52 cases (86.7%), more than 2.0 in 29 cases (48.3%) and more than 3.0 in 16 cases (26.7%) of 60 cases examined (Figure 1).

Intratumoural dThdPase concentrations are shown in Table 2. dThdPase levels were significantly associated with macroscopic types, microscopic types and the amount of tumour stroma. In the macroscopic type, amounts of dThdPase were significantly higher in type 1 than in types 0, 3, 4 and 5, while those in type 2 were greater than in types 0, 4 and 5. In the microscopic type, poorly differentiated and solid type had significantly higher concentrations of dThdPase than well or moderately differentiated tubular type, poorly differentiated and non-solid type, and signet ring cell type. In the amount of tumour stroma, dThdPase levels were significantly higher in the medullary type than in other types (Figure 2). Although there was no significant difference among the depth of invasion by ANOVA, dThdPase protein levels in cases with serosal invasion ($n$ = 31) were significantly higher than in cases without serosal invasion ($n$ = 43) (106.9 ± 98.9 and 67.2 ± 33.1, respectively, $P < 0.05$). There were no significant correlations between dThdPase and age, lymph node metastasis, peritoneal dissemination, lymphatic invasion, venous invasion and stages.

In the medullary type of the amount of tumour stroma, protein levels of dThdPase were positively correlated with the vertical diameter of the tumour (Table 3, Figure 3). The amounts of dThdPase were not significantly correlated with depth of invasion and the horizontal diameter of the tumour (Table 3).

**Table 2** Intratumoral concentrations of dThdPase

| | dThdPase (U/mg protein) | Statistics |
|---|---|---|
| Sex | | NS |
| Male ($n$ = 51) | 99.6 ± 91.0 | |
| Female ($n$ = 24) | 73.5 ± 47.9 | |
| Macroscopic type [a] | | $P < 0.05$ |
| 0 (Early gastric cancer, $n$ = 16) | 63.3 ± 31.2 | |
| 1 ($n$ = 5) | 181.9 ± 141.7 | |
| 2 ($n$ = 8) | 164.0 ± 154.0 | |
| 3 ($n$ = 14) | 103.0 ± 68.0 | |
| 4 ($n$ = 16) | 60.2 ± 31.1 | |
| 5 (Unclassified, $n$ = 15) | 69.3 ± 33.8 | |
| Histological type | | $P < 0.05$ |
| Papillary ($n$ = 1) | 56.4 | |
| Well differentiated tubular ($n$ = 8) | 58.8 ± 24.7 | |
| Moderately differentiated tubular ($n$ = 18) | 106.6 ± 94.8 | |
| Poorly differentiated and solid ($n$ = 10) | 172.3 ± 129.6 | |
| Poorly differentiated and non-solid ($n$ = 24) | 60.8 ± 32.3 | |
| Signet ring cell ($n$ = 6) | 63.2 ± 22.3 | |
| Mucinous ($n$ = 7) | 102.9 ± 76.2 | |
| Adenosquamous ($n$ = 1) | 117.7 | |
| Lymph node metastasis | | NS |
| Negative ($n$ = 25) | 70.8 ± 40.7 | |
| Positive ($n$ = 49) | 100.2 ± 93.3 | |
| Peritoneal dissemination | | NS |
| Negative ($n$ = 58) | 97.1 ± 85.8 | |
| Positive ($n$ = 17) | 71.2 ± 55.2 | |
| Depth of invasion | | NS |
| Mucosa ($n$ = 7) | 45.8 ± 21.6 | |
| Submucosa ($n$ = 9) | 78.1 ± 30.6 | |
| Muscularis propria ($n$ = 11) | 75.0 ± 40.2 | |
| Subserosa ($n$ = 4) | 58.3 ± 20.2 | |
| Serosa exposed ($n$ = 38) | 106.0 ± 102.8 | |
| Serosa infiltrating ($n$ = 5) | 114.0 ± 69.9 | |
| Lymphatic invasion | | NS |
| Negative ($n$ = 25) | 91.9 ± 98.3 | |
| Positive ($n$ = 48) | 89.3 ± 71.4 | |
| Venous invasion | | NS |
| Negative ($n$ = 48) | 81.9 ± 63.4 | |
| Positive ($n$ = 24) | 108.3 ± 108.5 | |
| Amount of stroma | | $P < 0.05$ |
| Medullary ($n$ = 17) | 145.4 ± 111.6 | |
| Intermediate ($n$ = 23) | 96.0 ± 86.3 | |
| Scirrhous ($n$ = 28) | 59.6 ± 27.1 | |
| Stage | | NS |
| Ia ($n$ = 15) | 60.7 ± 30.1 | |
| Ib ($n$ = 7) | 85.8 ± 44.8 | |
| II ($n$ = 7) | 73.5 ± 42.7 | |
| IIIa ($n$ = 15) | 117.2 ± 106.5 | |
| IIIb ($n$ = 11) | 135.2 ± 128.2 | |
| IVa ($n$ = 4) | 54.4 ± 31.8 | |
| IVb ($n$ = 15) | 79.4 ± 62.7 | |
| Recurrence ($n$ = 1) | 165.3 | |

[a]See footnote to Table 1 for classification.

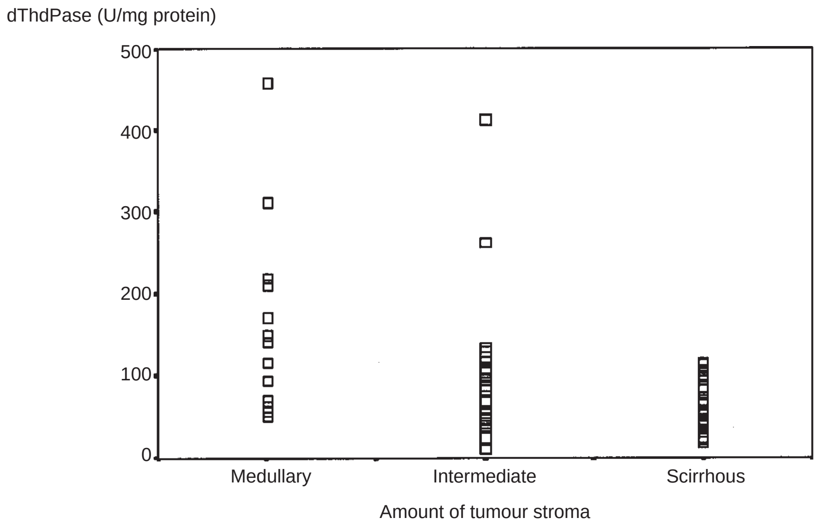

**Figure 2** dThdPase protein levels and the amount of stroma in cancer tissue

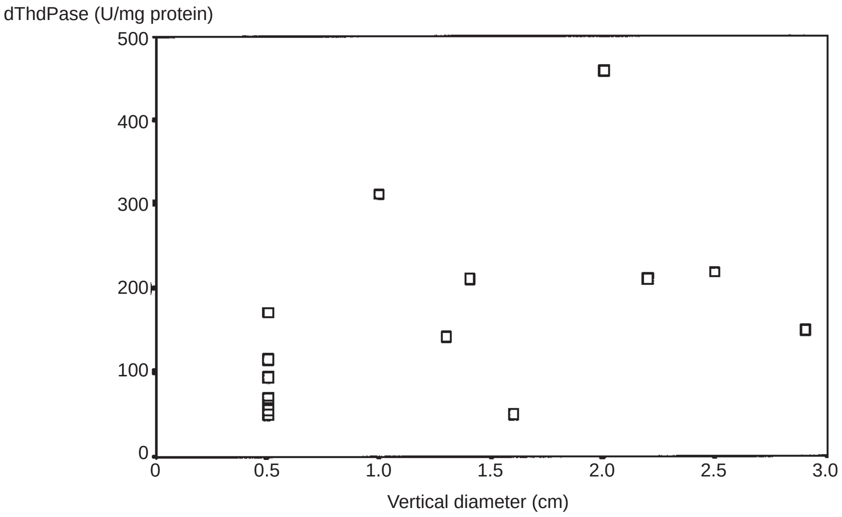

**Figure 3** dThdPase protein levels and the vertical diameter in the medullary type of the amount of stroma

**Table 3** Correlations between depth of invasion and maximal diameters in horizontal and vertical sections

| | *r* | *P*-value |
|---|---|---|
| All cases (*n* = 74) | | |
| Depth of invasion | 0.101 | 0.404 |
| Horizontal diameter | –0.04 | 0.973 |
| Vertical diameter | 0.146 | 0.226 |
| Medullary type (*n* = 17) | | |
| Depth of invasion | 0.468 | 0.058 |
| Horizontal diameter | 0.310 | 0.227 |
| Vertical diameter | 0.580 | 0.019 |

The normal gastric mucosal cells were not stained with anti-dThdPase antibody. Unequivocal staining of cytoplasm and nuclear compartment were observed in most of the carcinoma cells in all seven specimens with high dThdPase protein levels (Table 4, Figure 4), while in all cases with low dThdPase levels, most of the tumour cells were not stained (Table 4, Figure 5); there was a significant difference in the staining of carcinoma cells between these two groups by $\chi^2$ test. dThdPase expression was often observed in the tumour cells invading vessels (Figure 6). On the other hand, the expression on macrophage-like cells on the stromal cells was observed in all 14 cases with low dThdPase levels and in four of seven cases with high levels (Table 4, Figures 4 and 5).

### DISCUSSION

dThdPase has been reported to be increased in a variety of types of tumours (Kono et al, 1983; Nio et al, 1992). Carcinomas in the stomach contained higher amounts of dThdPase than non-neoplastic gastric mucosa (Yoshimura et al, 1990). As 5′-DFUR is metabolized to 5-FU by dThdPase (Miwa et al, 1987; Ishikawa et al, 1989), 5′-DFUR has been expected to be more specific and effective in tumour tissue than 5-FU. In this study, we demonstrated that dThdPase levels were increased in tumour tissue compared with those in normal mucosa in 86.7% of primary

 *© Cancer Research Campaign 1999*

**Table 4** Site of production and the concentrations of dThdPase

| | Tumour cells | Stromal cells |
|---|---|---|
| High contents (≥ 200 U/mg protein) | 7/7 | 4/7 |
| Medullary (*n* = 5) | 5/5 | 2/5 |
| Intermediate (*n* = 2) | 2/2 | 2/2 |
| Low contents (< 70 U/mg protein) | 0/14 | 14/14 |
| Medullary (*n* = 3)[a] | 0/3 | 3/3 |
| Intermediate (*n* = 4) | 0/4 | 4/4 |
| Scirrhous (*n* = 7) | 0/7 | 7/7 |

[a]All cases were early gastric cancer.

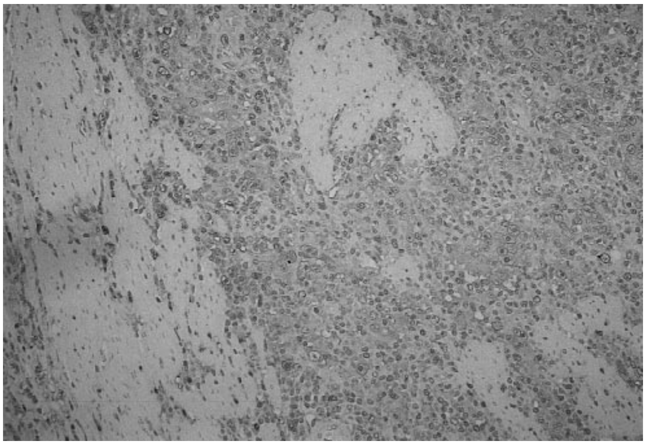

**Figure 4** Immunohistochemical staining for dThdPase (original magnification × 50). There is a strong staining of cytoplasm and nuclear compartment of the tumour cells gastric cancer patients; moreover, 48.3% of patients had more than two times the level of dThdPase in tumour tissue than appears in normal tissue. Higher T/N ratios of dThdPase activity are preferable in patients who are treated with 5′-DFUR, because of the probable high cytotoxicity against tumour tissue and the low toxicity against normal tissue. Determination of tissue levels of dThdPase would be useful for selecting the most appropriate patients for 5′-DFUR treatment.

To evaluate the characteristics of gastric carcinoma with high protein levels of dThdPase, clinicopathological findings were analysed. Our data demonstrated that dThdPase protein levels were increased in Borrmann types I and II macroscopically, in poorly differentiated and solid type histologically, in the medullary type of the tumour stroma, and in the tumour-invading serosa. In the medullary type of the amount of tumour stroma, dThdPase was positively correlated with the vertical diameter of the tumour. These data indicated that dThdPase is increased in advanced solid tumours in gastric cancer. Vascularization of advanced solid tumours is known to be poor and disordered, leading to insufficient perfusion and diffusion of nutrients to areas of the tumour (Griffiths and Stratford, 1997). This is thought to be a major reason for the existence of hypoxia, glucose depletion and low pH within solid tumours (Griffiths and Stratford, 1997). It was indicated that the expression of dThdPase was induced by hypoxia and low pH in human breast tumour cells (Griffiths et al, 1997). These mechanisms might be involved in higher amounts of dThdPase observed in advanced solid tumours in gastric cancer.

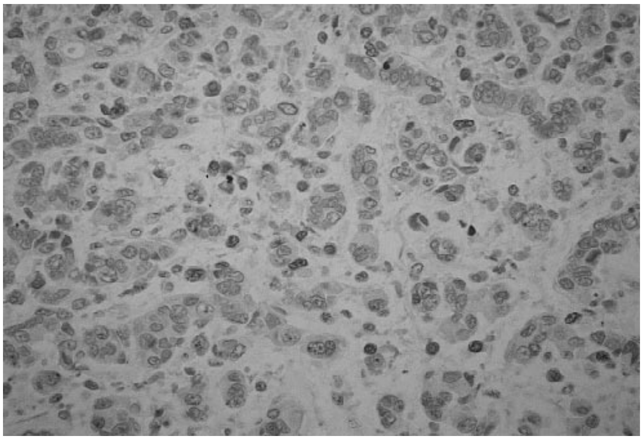

**Figure 5** Immunohistochemical staining for dThdPase (original magnification × 100). There is a strong staining in the stromal cells, but not in the tumour cells

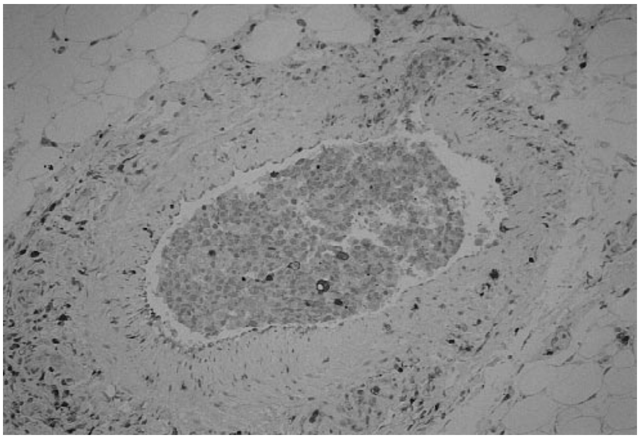

**Figure 6** Immunohistochemical staining for dThdPase in cancer cells invading vessels (original magnification × 40). There is a strong staining of tumour cells inside the vessels

Previously, only two investigators evaluated the expression of dThdPase in gastric cancer by immunohistochemistry (Maeda et al, 1996; Takebayashi et al, 1996a). It was demonstrated that dThdPase expression was detected in 42% of gastric cancer and observed in gastric carcinoma cells but not in normal mucosa (Takebayashi et al, 1996a). It was also indicated that dThdPase expression was detected in 60.8% of gastric cancer and mainly observed in tumour cells but also sometimes in endothelial cells, lymphocytes or macrophages invaded into tumour stroma (Maeda et al, 1996). On the other hand, in colon and breast cancer, the production of dThdPase by infiltrating cells has been investigated and found to contribute to overall tumour levels (Takebayashi et al, 1996b). As shown in this study, dThdPase expression was observed in not only tumour cells but also stromal cells, suggesting that production in both of these cells would contribute to the overall dThdPase protein content in gastric cancer tissue. Moreover, the expression of dThdPase in tumour cells was mostly observed in the advanced solid type with high dThdPase protein content. These data support our hypothesis that dThdPase production in carcinoma cells might be induced by the microenvironment.

© Cancer Research Campaign 1999

dThdPase in gastric cancer tissue was reported to be related to progression, metastasis and prognosis (Takebayashi et al, 1996a). It has also been indicated that dThdPase is associated with venous invasion, intratumoural microvessel count and hepatic metastasis, although that is not an independent prognostic factor in gastric cancer (Maeda et al, 1996). Our data did not indicate the relation of dThdPase levels with venous invasion and metastasis; however, dThdPase expression was often observed in the tumour cells invading vessels. It is likely that the evaluation by tumour cell staining observed by immunohistochemistry is different from the overall protein levels. dThdPase was not reported to be correlated with microvessel count in breast cancer (Fox et al, 1996). Fox et al (1996) suggested that dThdPase possibly involves in the early phase of angiogenesis as dThdPase is not mitogenic but chemotactic. In gastric cancer, hepatic metastasis was reported to be frequent in types 1 and 2 macroscopically, tubular type and poorly differentiated and solid type histologically, and medullary type of tumour stroma (Kito et al, 1981). As indicated in this study, dThdPase protein levels were increased in these types. Further investigation will be needed to clarify the role of dThdPase on hepatic metastasis in gastric cancer.

### REFERENCES

Cook AF, Holman MJ, Kramer MJ and Trown PW (1979) Fluorinated pyrimidine nucleoside. 3. Synthesis and antitumor activity of a series of 5′-deoxy-5-fluoropyrimidine nucleosides. *J Med Chem* **22**: 1330–1335

Eda H, Fujimoto K, Watanabe S, Ura M, Ura M, Hino A, Tanaka Y, Wada K and Ishitsuka H (1993) Cytokines induce thymidine phosphorylase expression in tumour cells and make them more susceptible to 5′-deoxy-5-fluorouridine. *Cancer Chemother Pharmacol* **32**: 333–338

Folkman J (1990) What is the evidence that tumors are angiogenesis dependent? *J Natl Cancer Inst* **82**: 4–6

Folkman J and Klagsbrun M (1987) Angiogenic factors. *Science* **235**: 442–447

Fox SB, Westwood M, Moghaddam A, Comley M, Turley H, Whitehouse RM, Bicknell R, Gatter KC and Harris AL (1996) The angiogenic factor, platelet derived endothelial cell growth factor thymidine phosphorylase is up-regulated in breast cancer epithelium and endothelium. *Br J Cancer* **73**: 275–280

Furukawa T, Yoshimura A, Sumizawa T, Haraguchi M and Akiyama S (1992) Angiogenic factor. *Nature* **356**: 668

Griffiths L and Stratford IJ (1997) Platelet-derived endothelial cell growth factor thymidine phosphorylase in tumor growth and response to therapy. *Br J Cancer* **76**: 689–693

Griffiths L, Dachs G, Bicknell R, Harris AL and Stratford IJ (1997) The influence of oxygen tension and pH on the expression of platelet-derived endothelial cell growth factor/thymidine phosphorylase in human breast tumor cells grown in vitro and in vivo. *Cancer Res* **57**: 570–572

Ishikawa F, Miyazono K, Hellmen U, Drexler H, Wernstedt C, Hagiwara K, Usuki K, Takaku E, Risau W and Heldin C-H (1989) Identification of angiogenic activity and the cloning and expression of platelet-derived endothelial cell growth factor. *Nature* **338**: 557–562

Ishitsuka H, Miwa M, Takemoto K, Fukuoka K, Itoga A and Maruyama HD (1980) Role of uridine phosphorylase for anti-tumor activity of 5′-deoxy-5-fluorouridine. *Gann* **71**: 112–123

Japanese Research Society for Gastric Cancer (1995) Japanese Classification of Gastric Carcinoma, First English Edition. Kanehara: Tokyo

Kito T, Yamada E, Miyaishi S, Nakazato H, Kato H, Takagi M, Yasue T, Kato T, Morimoto S, Yamauchi S, Suchi T, Sato C and Suzuki R (1981) Prognosis and pathological type in advanced gastric cancer (in Japanese). *Geka* **43**: 1041–1046

Kono A, Hara Y, Sugata S, Karube Y, Matsushima Y and Ishituka H (1983) Activation of 5′-deoxy-5-fluorouridine by thymidine phosphorylase in human tumors. *Chem Pharm Bull Tokyo* **31**: 175–178

Maeda K, Chung YS, Ogawa Y, Ogawa Y, Takatsuka S, Kang SM, Ogawa M, Sawada T, Onoda N, Kato Y and Sowa M (1996) Thymidine phosphorylase/platelet-derived endothelial cell growth factor expression associated with hepatic metastasis in gastric carcinoma. *Br J Cancer* **73**: 884–888

Miwa M, Nishimura J, Kamiyama T and Ishitsuka H (1987) Conversion of 5′-deoxy-5-fluorouridine to 5-FU by pyrimidine nucleoside phosphorylases in normal and tumor tissues from rodents bearing tumors and cancer patients. *Jpn J Cancer Res* **14**: 2924–2929

Miyazono K, Okabe T, Urabe A, Takaku F and Heldin CH (1987) Purification and properties of an endothelial cell growth factor from human platelets. *J Biol Chem* **262**: 4098–4103

Nio Y, Kimura H, Tsubono M, Tseng CC, Kawabata K, Masai Y, Hayashi H, Meyer C, Fukumoto M and Tobe T (1992) Anti-tumor activity of 5′-deoxy-5-fluorouridine in human digestive organ cancer xenografts and pyrimidine nucleoside phosphorylase activity in normal and neoplastic tissues from human digestive organs. *Anticancer Res* **12**: 1141–1146

Nishida M, Hino A, Kazushige M, Matsumoto T, Yoshikubo T and Ishitsuka H (1996) Preparation of anti-human thymidine phosphorylase monoclonal antibodies useful for detecting the enzyme levels in tumor tissues. *Biol Pharm Bull* **19**: 1407–1411

Takebayashi Y, Miyadera K, Akiyama S, Hokita S, Yamada K, Akiba S, Yamada Y, Sumizawa T and Aikou T (1996a) Expression of thymidine phosphorylase in human gastric carcinoma. *Jpn J Cancer Res* **87**: 288–295

Takebayashi Y, Yamada K, Miyadera K, Sumizawa T, Furukawa T, Kinoshita E, Aoki D, Okimura H, Yamada Y, Akiyama S and Aikou T (1996b) The activity and expression of thymidine phosphorylase in human solid tumors. *Eur J Cancer* **32**: 1227–1232

Yoshimura A, Kuwazuru Y, Furukawa T, Yoshida H, Yamada K and Akiyama S (1990) Purification and tissue distribution of human thymidine phosphorylase; high expression in lymphocytes, reticulocytes and tumors. *Biochim Biophys Acta* **1034**: 107–113

*© Cancer Research Campaign 1999*
